# Seed‐cone architecture of *Pararaucaria utahensis* Gee, Howell et Dayvault sp. nov. (Cheirolepidiaceae) from the Upper Jurassic Morrison Formation of Utah, USA

**DOI:** 10.1002/ajb2.70264

**Published:** 2026-09-17

**Authors:** Carole T. Gee, Mariah M. Howell, Richard D. Dayvault

**Affiliations:** ^1^ Bonn Institute of Organismic Biology, Division of Paleontology University of Bonn Nussallee 8 53115 Bonn Germany; ^2^ Utah Field House of Natural History State Park Museum 496 East Main Street Vernal 84078 Utah USA; ^3^ Senckenberg – Leibniz Institution for Biodiversity and Earth System Research Senckenberg Forschungsinstitut und Naturmuseum Frankfurt Senckenberganlage 25 60325 Frankfurt am Main Germany; ^4^ 2153 Redcliff Circle Grand Junction Colorado 81507 USA

**Keywords:** Cheirolepidiaceae, cone phyllotaxis, cone phyllotaxy, fossil conifer seed cone, microCT, microtomography, Morrison Formation, *Pararaucaria utahensis*, Upper Jurassic paleobotany

## Abstract

**Premise:**

Although *Pararaucaria* seed cones were first described 90 years ago, only in the last dozen years have we discovered the greater extent of the species diversity and paleogeographic range of the genus. *Pararaucaria* is now known across the mid‐latitudes of the Lower Jurassic to Lower Cretaceous in the northern and southern hemispheres. Here we describe a new species, *Pararaucaria utahensis* Gee, Howell et Dayvault sp. nov. from the Upper Jurassic Morrison Formation in Utah, United States.

**Methods:**

We studied the gross morphology of 12 permineralized cones and used micro‐computed tomography (microCT) and three‐dimensional visualization to investigate cone architecture and internal structure nondestructively.

**Results:**

Imaging revealed a slender, cylindrical, woody cone axis with a small pith. The scale and bract traces separate near the axis, with the scale trace becoming flattened distally. Three parastichy spirals wrap around the cone, and each bears six pairs of seeds. The ontogenetic development of seeds and bract/scale complexes in this cone species is generally counterclockwise, from apex to base. In nearly all cases, there are two seeds per bract/scale complex. The cone peduncle of one cone bears small *Brachyphyllum*‐type scale leaves.

**Conclusions:**

In addition to the formal description of a seventh global species of *Pararaucaria* and the second species in North America, our study of *P. utahensis* provides clear megafossil evidence for Cheirolepidiaceae in the Upper Jurassic Morrison Formation of western North America. However, the scanty evidence available for the cheirolepidiaceous seed cones in comparison to widespread pollen remains puzzling.

The family Cheirolepidiaceae is a major group of conifers that thrived during the Mesozoic, from the Late Triassic to the Late Cretaceous (Alvin, 1982; Watson 1988; Mendes et al., 2025), although cheirolepidiaceous remains have also been found extending into the Paleocene (Barreda et al., 2012). Cheirolepidiaceae were a major component of global forests, alongside Araucariaceae and Podocarpaceae (Gee et al., 2020), but were especially common in warm habitats at low to mid‐latitudes (e.g., Alvin, 1982; Watson, 1988; Marmi et al., 2023). The family commonly dominated coastal wetlands, particularly brackish or halophytic environments (e.g., Francis, 1983, 1984; Marmi et al., 2023), but also grew in hot, dry, and seasonally arid environments (Axsmith and Jacobs, 2005).

Cheirolepidiaceae had the widest diversity in its morphology, habit, and habitat among all conifers, ancient or living (Watson, 1988). For example, the family exhibited a broad spectrum of growth forms, ranging from small‐stem succulents (Watson, 1977) to huge trees (Francis, 1983; Axsmith and Jacobs, 2005; Falaschi et al., 2011; Steart et al., 2014, 2023). The most recognizable and widespread evidence of Cheirolepidiaceae is its unique pollen grain known as *Classopollis*, which is sometimes considered the only unifying character of the family. It should also be noted, however, that cheirolepidiaceous leaves are also distinctive in having a cuticle with sunken stomata and protruding papillae (Alvin, 1982; Watson, 1977, 1988; Taylor et al., 2009). The cheirolepidiaceous seed cones are lesser known and include *Hirmeriella*, *Pararaucaria*, and the newly recognized genus *Arkansia* (Andruchow‐Colombo and Matsunaga, 2026). Unlike many fossil plants where only isolated cones or leaves are found, *Hirmeriella*, specifically *H. muensteri* (Schenk) Jung, is one of the few Mesozoic conifers that has been reconstructed as a whole plant, as its seed cones, pollen cones, pollen, and foliage have been unequivocally linked to one another (Barbacka et al., 2007). *Hirmeriella* cones are thus considerably better known than those of *Pararaucaria* or *Arkansia*.

*Pararaucaria* cones have remained enigmatic in terms of their structure and familial affinity for nine decades after they were first named by Wieland (1935). It was not until excellently preserved, permineralized cones of the type species, *Pararaucaria patagonica* Wieland emend. Escapa, Rothwell, Stockey et Cúneo, were described in 2012 that a deeper understanding of the distinctive “pocket‐forming tissue” and the three‐lobed distal end of the ovulate scale was achieved (Escapa et al., 2012). Since then, several new species of *Pararaucaria* have been formally described: *Pararaucaria delfueyoi* Escapa, Cúneo, Rothwell et Stockey (2013), *P. carrii* Stockey et Rothwell (2013), *P. collinsonae* Steart, Spencer, Kenrick, Needham et Hilton (Steart et al., 2014), *P. taquetrensis* Escapa et Leslie (2017), and *P. laiyangensis* Jin P. H. et Sun B. N. (Jin et al., 2023).

Each successive study on a new species of *Pararaucaria* has yielded valuable information on the genus, as well as on the individual species. The new information gained depended in great part on the mode of fossilization of the cones. Permineralized cones lend themselves to anatomical investigation (Stockey, 1977; Escapa et al., 2012, 2013; Stockey and Rothwell, 2013; Steart et al., 2014), while coalified compressions can provide cuticular details of any associated leaves (Jin et al., 2023). Studies on *Pararaucaria* cones found preserved as both compressions and permineralizations can help us to understand the different appearance of the same species in different preservational modes (Escapa and Leslie, 2017). Modern methodological approaches, such as synchrotron‐radiation microtomography or microCT imaging, can elucidate the inner structures of the cone with no destruction or damage to the original specimens (Gee et al., 2014; Steart et al., 2014; M. M. Howell, unpublished data).

In 2014, a new type of conifer seed cone previously unknown from the Morrison Formation was referred to as Morphotype 5, alongside four more new cone morphotypes (Gee et al., 2014). Morphotype 5 was described as a small, globose to cylindrical, ovulate cone pertaining to the genus *Pararaucaria* of the extinct conifer family Cheirolepidiaceae. Further details, such as its taxonomic assignment, were not given at that time. However, since then, nine new cones of Morphotype 5 have been discovered in the Morrison Formation, for a total of 12 cones, making possible a more complete delineation of this species. Here we name cones of this morphological type as a new species, *Pararaucaria utahensis* Gee, Howell et Dayvault and describe in more detail the cone architecture and phyllotaxis of the *P. utahensis*, which was made possible with the application of high‐resolution X‐ray microcomputed tomography integrated with 3D reconstruction. *Pararaucaria utahensis* is the seventh species assigned to the genus *Pararaucaria*, the second species described from North America, and the first one from the Upper Jurassic Morrison Formation of the western United States.

## GEOLOGICAL SETTING

**Figure 1:**
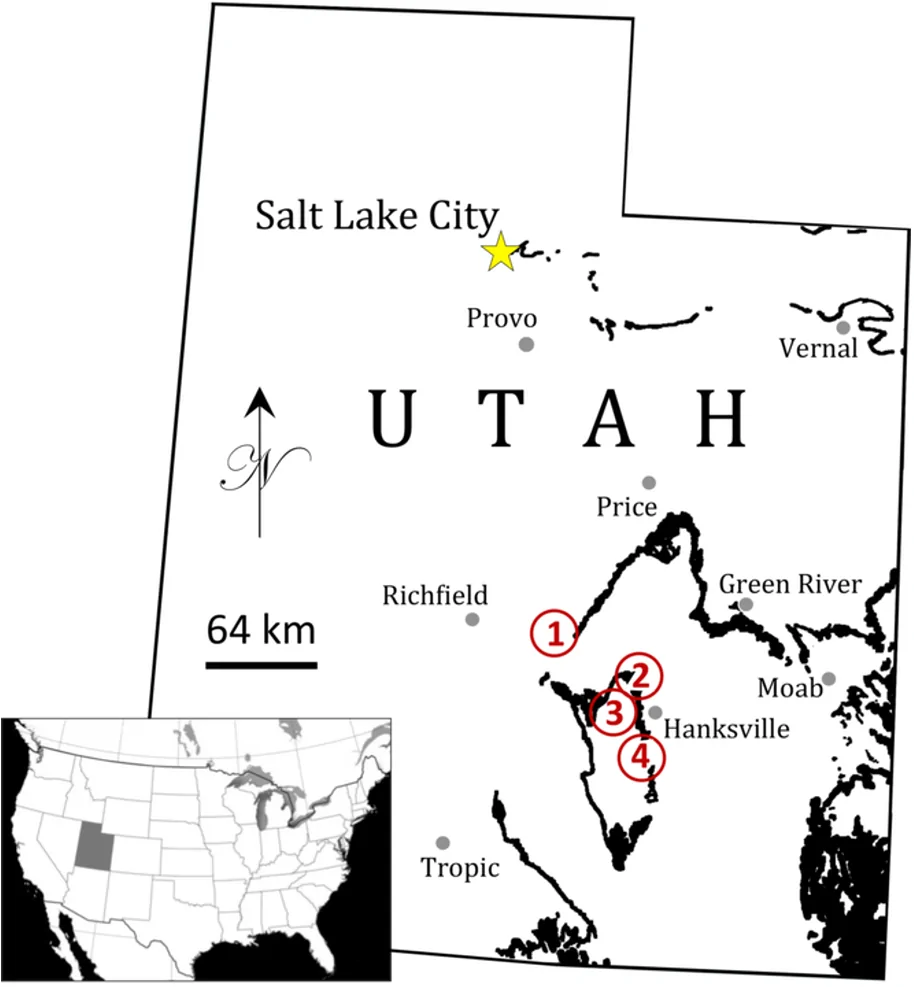
Map of Utah marked with the four fossil cone localities: (1) Fremont Junction site, (2) Little Wild Horse site, (3) Caineville Wash site, and (4) the site on the north slope of Mt. Ellen in the Henry Mountains. Irregular black lines and shapes depict the outcrops of the Upper Jurassic Morrison Formation; map courtesy of Ken Carpenter (University of Colorado Boulder). Inset on the lower left: Map of the USA, with the state of Utah marked in gray (modified from Nations Online Project, https://www.nationsonline.org/oneworld/usa__blank_map.htm).

The fossil conifer cones under study here all originate from the Upper Jurassic Morrison Formation in Utah, United States (Figure 1). The Morrison Formation is a laterally widespread formation in the Western Interior of North America extending from Montana to New Mexico. However, the formation crops out only on the eastern side of the state of Utah. The fossil cones come from four localities in central Utah: the Fremont Junction site, the Little Wild Horse site near Goblin Valley State Park, the Caineville Wash site, and the site on the north slope of Mt. Ellen in the Henry Mountains. The fossil cones were gathered by various collectors over the course of decades (Table 1). In all cases, fossil material was recovered from the Brushy Basin Member (Dayvault and Hatch, 2007) in the uppermost Morrison Formation, which has been dated in Wayne County as 152–151 million years old and in Emery County as 152–150 million years old (Trujillo and Kowallis, 2015).

**Table 1:** Collection information on the 12 cones of *Pararaucaria utahensis* Gee, Howell et Dayvault sp. nov. studied here.

CG inventory number	Museum specimen number, Current repository	Locality	Collector
CG‐029	BYU 53602, Brigham Young University Museum of Paleontology, Provo, Utah, USA	Fremont Junction site, Sevier County, Utah, USA	Homer Behunin
CG‐043a, b	Dayvault Collection in Bonn, Germany	Caineville Wash site, Wayne County, Utah, USA	Richard D. Dayvault
CG‐044a, b	Dayvault Collection in Bonn, Germany	Caineville Wash site, Wayne County, Utah, USA	Richard D. Dayvault
CG‐068	Gee Collection in Bonn, Germany	Little Wild Horse site, near Goblin Valley State Park, Emery County, Utah, USA	Carole T. Gee and Richard D. Dayvault
CG‐074	A1, Corrales Collection in Clearview, Utah, USA	Little Wild Horse site, near Goblin Valley State Park, Emery County, Utah, USA	Nathan Corrales
CG‐075	A2, Corrales Collection in Clearview, Utah, USA	Little Wild Horse site, near Goblin Valley State Park, Emery County, Utah, USA	Nathan Corrales
CG‐078	B1, Corrales Collection in Clearview, Utah, USA	Little Wild Horse site, near Goblin Valley State Park, Emery County, Utah, USA	Nathan Corrales
CG‐081	Dayvault Collection in Bonn, Germany	Sands Seep, north slope of Mt. Ellen, Henry Mountains, Garfield County, Utah, USA	Harold Ekker and H. Steven Hatch
CG‐082	Dayvault Collection in Bonn, Germany	Moose's Knob, Mt. Ellen, Henry Mountains, Garfield County, Utah, USA	Harold Ekker and H. Steven Hatch
CG‐085	BYU 1781/20628, Brigham Young University Museum of Paleontology, Provo, Utah, USA	Fremont Junction site, Sevier County, Utah, USA	Homer and Joanne Behunin and James A. Jensen (Chandler, 1966)
CG‐092	UF 19577‐82985, Florida Museum of Natural History (Paleobotany)	Little Wild Horse site, near Goblin Valley State Park, Emery County, Utah, USA	Richard D. Dayvault
CG‐097	UF 19571‐84109, Florida Museum of Natural History (Paleobotany)	Little Wild Horse site, near Goblin Valley State Park, Emery County, Utah, USA	Richard D. Dayvault

All fossil cones under study here are silicified. In a few cones, some tissues have been preserved and at times, anatomical structure can be observed. Other cones are agatized and do not show any internal details.

## MATERIALS AND METHODS

### Study material

Fossil cones from several collections were assembled for this study (Table 1). These collections are deposited at the Division of Paleontology, Bonn Institute of Organismic Biology, University of Bonn (Bonn, Germany); the Paleobotanical Collections, Museum of Paleontology, Brigham Young University (Provo, UT, USA); the Paleobotany Collection at the Florida Museum of Natural History (Gainesville, FL, USA; and the Corrales collection (Clearfield, UT, USA). To facilitate recordkeeping during the ongoing study of these cones, we assigned an inventory number with the prefix “CG” to each cone.

### Light microscopy

A Leica S APO zoom microscope was used for stereomicroscopy of the fossil specimens and digital images were taken and processed with the integrated measurement software ImageAccess easyLab 7 (Version 1992–2007, iMagic, Glattburg, Switzerland). Longitudinal sections were studied in the few cones that had been cut longitudinally and polished. Measurements were taken with digital calipers (Mitutoyo 700‐103B LCD; Mitutoyo Corp., Kawasaki, Japan).

### MicroCT and 3D visualization

High‐resolution X‐ray microcomputed tomography (microCT) was applied to understand the three‐dimensional nature of the phyllotaxis of the cones in a nondestructive manner (Gee, 2013; Gee et al., 2014). MicroCT scans were made of all cones with the 240 kV/320 W microfocus tube of a Phoenix V|tome|x S 240D CT scanner coupled with scanning software phoenix datos|x, version 2.7.0 (General Electric Measurement and Control Solutions, Wunstorf, Germany), in the Division of Paleontology at the University of Bonn, Germany. The resulting projections were converted into orthoslices in image stacks with VG Studio Max (version 3.2; Volume Graphics, Heidelberg, Germany) in three planes of section—one transverse and two longitudinal planes. The orthoslice image stacks served as the basis of the 3D reconstruction created with the visualization and analysis software Avizo (version 8.1.1; FEI Visualization Sciences Group, Düsseldorf, Germany). Single orthoslices were also selected from the image stacks for image analysis and enhanced with Preview (version 11.0) on an iMac (OS Sonoma 14.5; Apple, Cupertino, CA, USA).

### Terminology

The terminology for plant tissues and structures follows the definitions given by Escapa et al. (2012).

## RESULTS

### Systematic paleobotany

***Order***—Coniferales Florin

***Family***—Cheirolepidiaceae Takhtajan

***Genus***—*Pararaucaria* Wieland emend. Escapa, Rothwell, Stockey et Cuneo, 2012

***Species***—*Pararaucaria utahensis* Gee, Howell et Dayvault sp. nov.

#### Previously published specimens

*Sequoia* sp.: Chandler (1966), pl. 8, figure 75, 76 (original material examined by C. T. Gee). Note: pl. 8, figure 77–79 excluded here.

Small, siliceous cone with large seeds: Daniels and Dayvault (2006), p. 302, left column, center photograph.

Small, equant siliceous cones showing exposed seeds: Daniels and Dayvault (2006), p. 302, center column, top photograph.

Small, equant siliceous cones with exposed seeds: Dayvault and Hatch (2007), p. 386, figure 9.

Longitudinally cut, colorful cone fragment showing large seeds: Dayvault and Hatch (2007), p. 387, figure 10.

Morphotype 5: Gee et al. (2014), figures 2e, 3d, 3e, 4e.

Cheirolepidiaceae: Gee et al. (2020), p. 168, figure 6.7e.

#### Species diagnosis

Ovoid, obovoid, or 3D oblong cones; smallest cones 18 mm long × 17 mm wide; largest cones 30 mm long × 20 mm wide. Cone axis slender with small‐diameter pith bearing helically arranged bract/scale complexes, each consisting of a large ovuliferous scale, subtended by an equally broad bract that is free from the ovuliferous scale. The two traces that either run into the bract or into the scale complex separate at their point of departure from the cone axis. Most scales with two seeds; at base of cone, seeds can occur singly or appear misshapen. Seeds inverted, obovate, up to 5 mm long, 2.5 mm wide, positioned at the base of the ovuliferous scale. Paired seeds with their facing sides flat‐edged and parallel to one another, enclosed together in a pocket‐forming tissue. Cone peduncle at cone base slender and woody, bearing short, triangular, helically arranged *Brachyphyllum*‐type scale leaves.

#### Etymology

The specific epithet *utahensis* honors the state of Utah, United States, where these cones were first discovered and have only been found up to now.

#### Holotype

**Figure 2:**
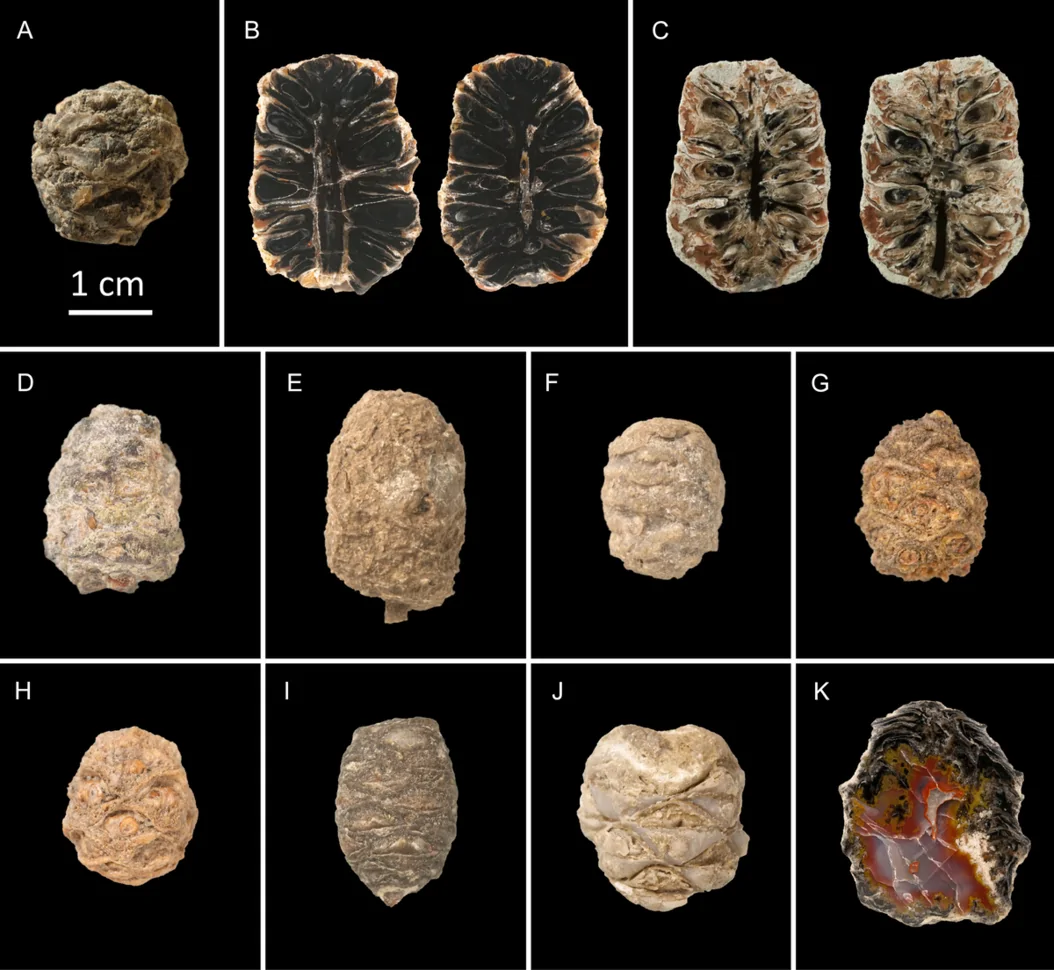
Eleven of the 12 seed cones of *Pararaucaria utahensis* Gee, Howell et Dayvault described here. The scale bar in A applies to all cones. The twelfth cone (CG‐078) is shown enlarged in Figure 3A, B. (A) Locality: Fremont Junction site, Sevier County; holotype in lateral surface view (CG‐029). (B, C) Caineville Wash site, Wayne County, cut and polished in longitudinal median section to form part and counterpart. B: CG‐043a, b. C: CG‐044a, b. (D–F) Locality: Little Wild Horse site, near Goblin Valley State Park, Emery County; in lateral surface view. D: CG‐068. E: CG‐074. F: CG‐075. (G) Locality: Sands Seep, north slope of Mt. Ellen, Henry Mountains, Garfield County; in lateral surface view (CG‐081). (H) Locality: Moose's Knob, Mt. Ellen, Henry Mountains, Garfield County; in lateral surface view (CG‐082). (I) Locality: Fremont Junction site, Sevier County, in lateral surface view (CG‐085). (J) Locality: Presumably from the Little Wild Horse site, near Goblin Valley State Park, Emery County; in lateral surface view (CG‐092). (K) Locality: Little Wild Horse site, near Goblin Valley State Park, Emery County; cut and polished in longitudinal median section to show internal agatization (CG‐097).

Paleobotanical Collections of the Museum of Paleontology at Brigham Young University in Provo, Utah, USA; inventory number 53602 (CG‐029), figured here as Figures 2A, 4A, 5A–E, Appendix S1.

#### Referred material

CG‐029, −043a, −043b, −044a, −044b, −068, −074, −075, −078, −081, −082, −085, −092, −097.

#### Stratigraphy and age

Brushy Basin Member of the Upper Jurassic Morrison Formation.

### Description

#### Gross morphology

Conifer seed cones spherical in form (Table 2), varying slightly from ovoid to obovoid (CG‐029; Figure 2A), to ellipsoid or more 3D oblong in shape (CG‐043a, b; CG‐044a, b, Figure 2B, C). The smallest cone known (CG‐029, Figure 2A) is 18 mm long and 17 mm wide at its widest point, while the largest cone (CG‐044, Figure 2C) is 30.5 mm long and 20.6 mm wide. Most specimens are lightly flattened longitudinally and abraded on the surface. The outer surface of some specimens shows a pattern of relatively large, broadly rhomboidal structures that represent the spirally arranged bract/scale complexes. One such rhomboidal structure measures 11 × 5 mm on the smallest cone (CG‐029, Figure 2A) and 13 × 8 mm on the largest specimen (CG‐044, Figure 2C). In these cases, the woodier bract in the lower half of the bract/scale complex is commonly better preserved, while the ovuliferous scale in the upper half is more highly abraded.

**Table 2:** Characteristics of the individual cones of *Pararaucaria utahensis* Gee, Howell et Dayvault. ø = diameter, CW = clockwise, CCW = counterclockwise, Indet. = indeterminate.

CG inventory number	3D shape	Max. height (mm)	Max. width (mm)	Seeds per bract/scale	ø Cone axis (mm)	Parastichy	Ontogenetic spiral (CW or CCW)	Figure or Appendix (S)
CG‐029	Obovoid	18.0	17.0	Normally 2	2.7	2,3	CCW	2 A, 4 A, 5A–E, S1
CG‐043a, b	3D oblong	27.7	19.1	Indet.	4.2 near base	2,3	CW	2B
CG‐044a, b	3D oblong	30.5	20.6	Indet.	1.6	2,3	Indet.	2 C
CG‐068	Ovoid	22.0	16.5	2	2.8	2,3	CCW	2D, S2
CG‐074	Ovoid	29.6	18.2	Indet.	3.0 × 3.4, avg. 3.2	Indet.	Indet.	2E
CG‐075	Ellipsoid	21.1	16.9	Indet.	Indet.	Indet.	Indet.	2 F
CG‐078	Obovoid	21.1	17.5	Indet.	6.4 × 5.2	2,3	CCW	3 A, B, S3
CG‐081	Ovoid	21.1	15.4	2	1.3	2,3	CCW	2 G
CG‐082	Ovoid	17.5	15.1	2	1.6	2,3	Indet.	2H, 4B
CG‐085	Obovoid^a^	21.1	14.6	Indet.	1.9	2,3	CCW	2I
CG‐092	Widely obovoid	23.1	20.9	2	3.1	2,3	Indet.	2 J
CG‐097	Ovoid	26.5	21.6	Indet.	Indet.	2,3	Indet.	2 K

^a^ Cone shape strongly distorted through abrasion.

#### Cone axis and pith

Polished median longitudinal sections of the largest two cones show a central, slender cone axis that is more or less uniform in width from the cone base to apex. In the smaller of the two larger cones (CG‐043), the central cone axis is widest near cone base, measuring up to 4.1 mm wide (Figure 2B). The pith within the cone axis is narrow and generally uniform in diameter, but widens slightly toward the cone base, measuring ca. 2.8 mm wide on the same cone (CG‐043).

#### Bract/scale complexes

**Figure 3:**
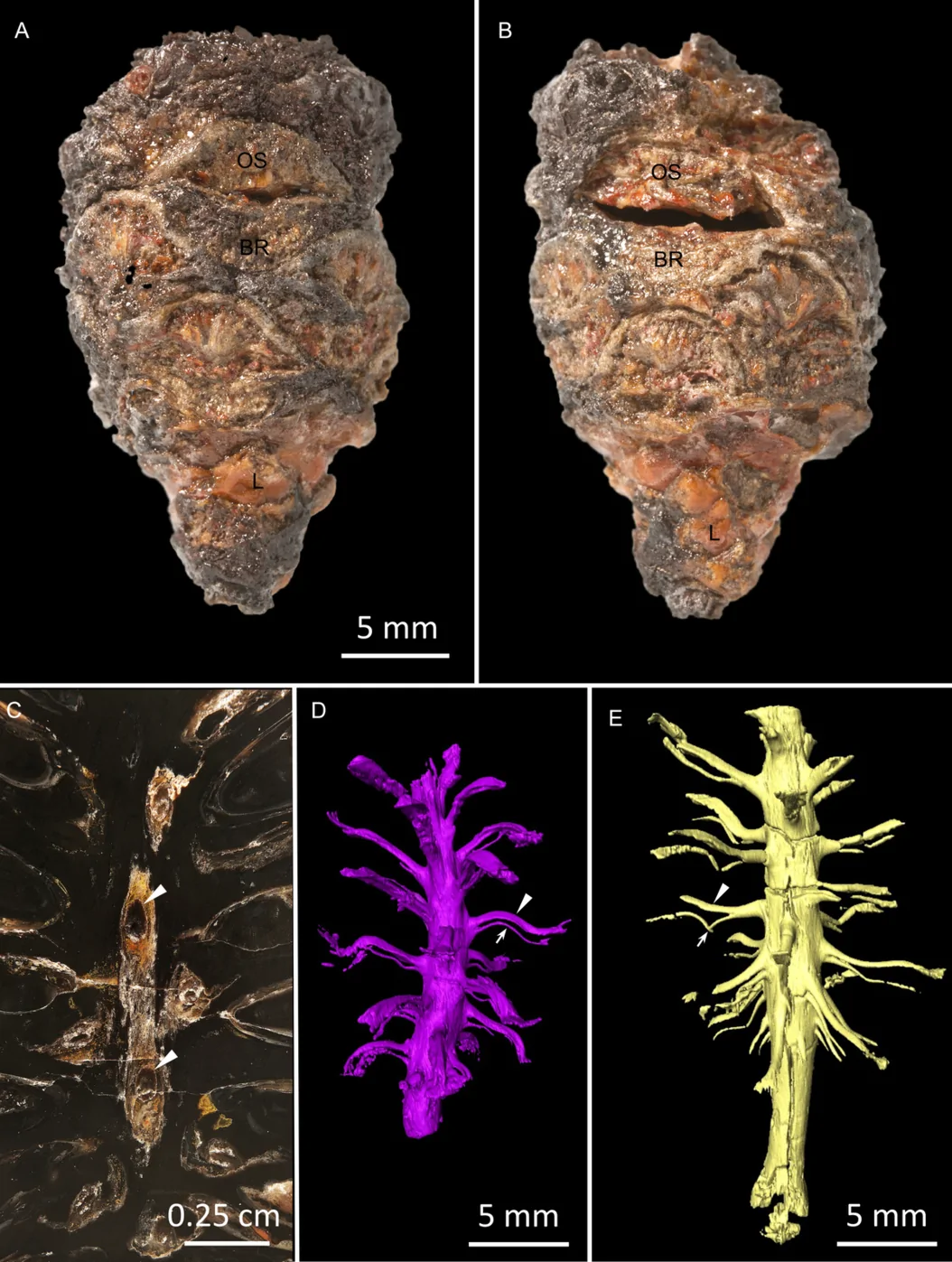
Close‐up images of *Pararaucaria utahensis* Gee, Howell et Dayvault sp. nov., showing the cone surface, the bract/scale trace scars on the cone axis, and separation of the bract/scale traces in 3D space. (A, B) Locality: Little Wild Horse site, near Goblin Valley State Park, Emery County, same cone in two lateral surface views (CG‐078). Note the gap between the ovuliferous scale (OS) and bract (BR) showing their unfused nature, as well as the *Brachyphyllum* leaves (L) on the cone pedicel. (C) Close‐up of the right cone in Figure 2B (CG‐043b), showing the cross section through two oval‐shaped, fused bract/scale traces on the surface of the cone axis (arrowheads). (D) 3D cone axis and vascular traces in cone CG‐068, imaged with microCT and segmented with Avizo, showing the slender central cone axis and the trace that divides into two and extends into the ovuliferous scale or bract, respectively (arrowhead, arrow; see Appendix S2 for animated version). (E) 3D segmented cone axis and vascular traces in cone CG‐078, imaged with microCT and segmented with Avizo, showing the slender central cone axis and the trace that divides into two and extends into the ovuliferous scale or bract, respectively (arrowhead, arrow; see Appendix S3 for animated version). The bract trace can change direction abruptly (arrow).

The bract/scale complexes mid‐cone arise nearly perpendicular to the cone axis (Figure 2B, C). The angle of attachment of the bract/scale complexes gradually becomes more acute toward the cone apex and more obtuse toward the cone base. The paired bract and ovuliferous scale are free for most of their length. At the cone surface, the paired ovuliferous scale and bract are broadly rhomboidal. They are virtually the same size and shape, with a gap between them that show their nonfused nature (Figure 3A, B).

#### Bract/scale traces

Each bract/scale trace appears as a single structure at its origin at the cone axis (Figure 3C). It is oval in outline and measures 1.2 × 0.8 mm (CG‐043b; Figure 3C, arrowheads). The trace separates soon thereafter into an upper scale trace (Figure 3D, E, arrowheads) and a lower bract trace (Figure 3D, E, arrows). As it continues distally into the ovuliferous scale, the scale trace becomes flattened in shape, which would look band‐shaped and lightly concave‐down in a transverse section (Figure 3E, arrowhead; Appendices S2, S3). It becomes much wider than the bract trace. The bract trace, on the other hand, more or less retains its size and shape during its course into the bract. In a transverse section, it would appear circular, but it can abruptly change its course of direction (Figure 3E, arrow).

#### Seeds

**Figure 4:**
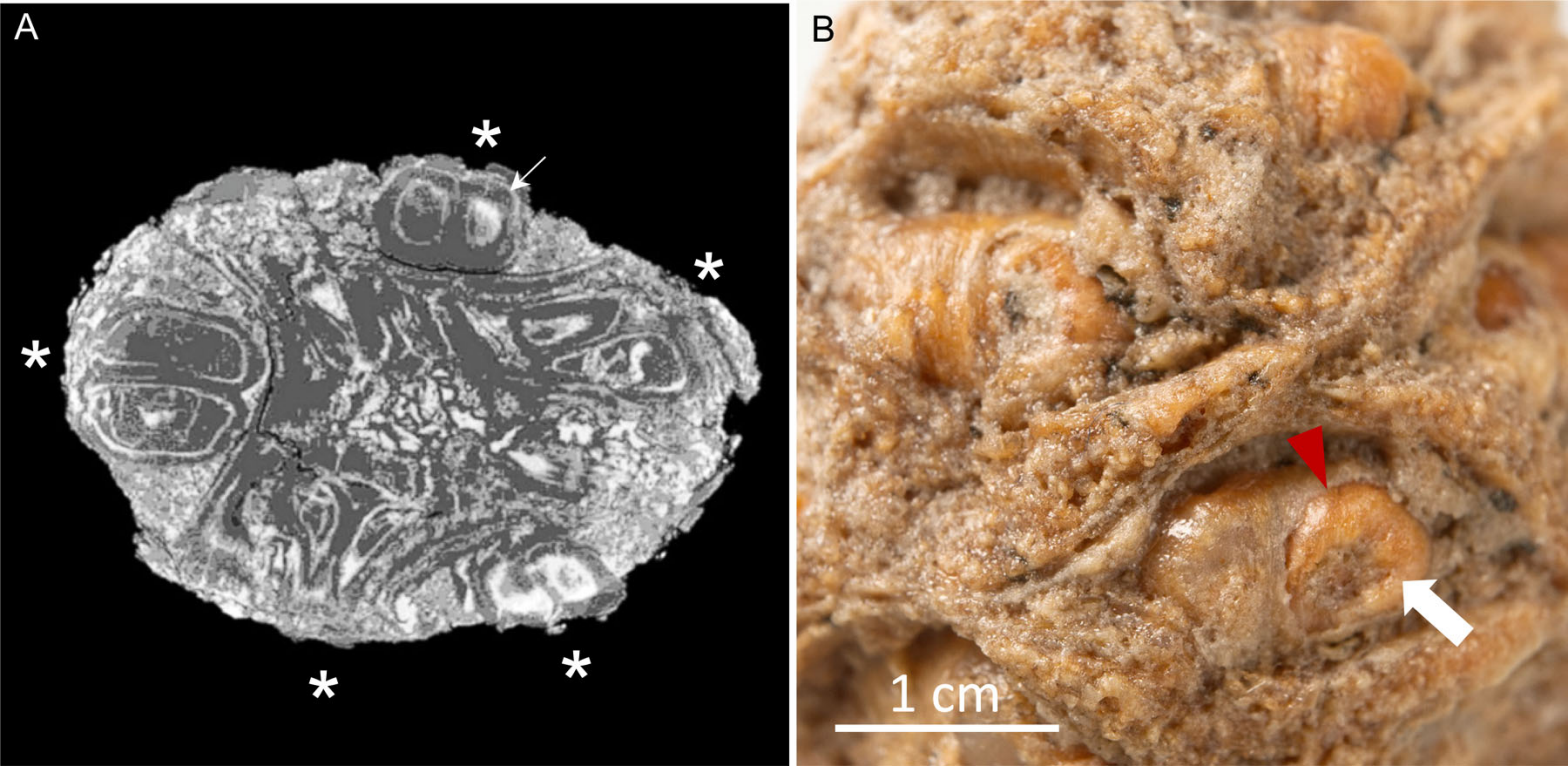
Details of the seeds and pocket‐forming tissue in the seed cones of *Pararaucaria utahensis* Gee, Howell et Dayvault sp. nov. (A) MicroCT orthoslice of cone CG‐029 in cross section, section 330/835. Note the five pairs of seeds per bract/scale complex observed at this level, each pair marked with an asterisk (*). The outline of the pocket‐forming tissue (arrow) is apparent in the top pair of seeds. (B) Detail of cone CG‐082 in Figure 2H, highlighting the two seeds of one surface‐abraded bract/scale. The pocket‐forming tissue was torn off the seed on the right (white arrow); the red arrowhead marks the edge of the torn pocket‐forming tissue, which still covers the seed on the left.

**Figure 5:**
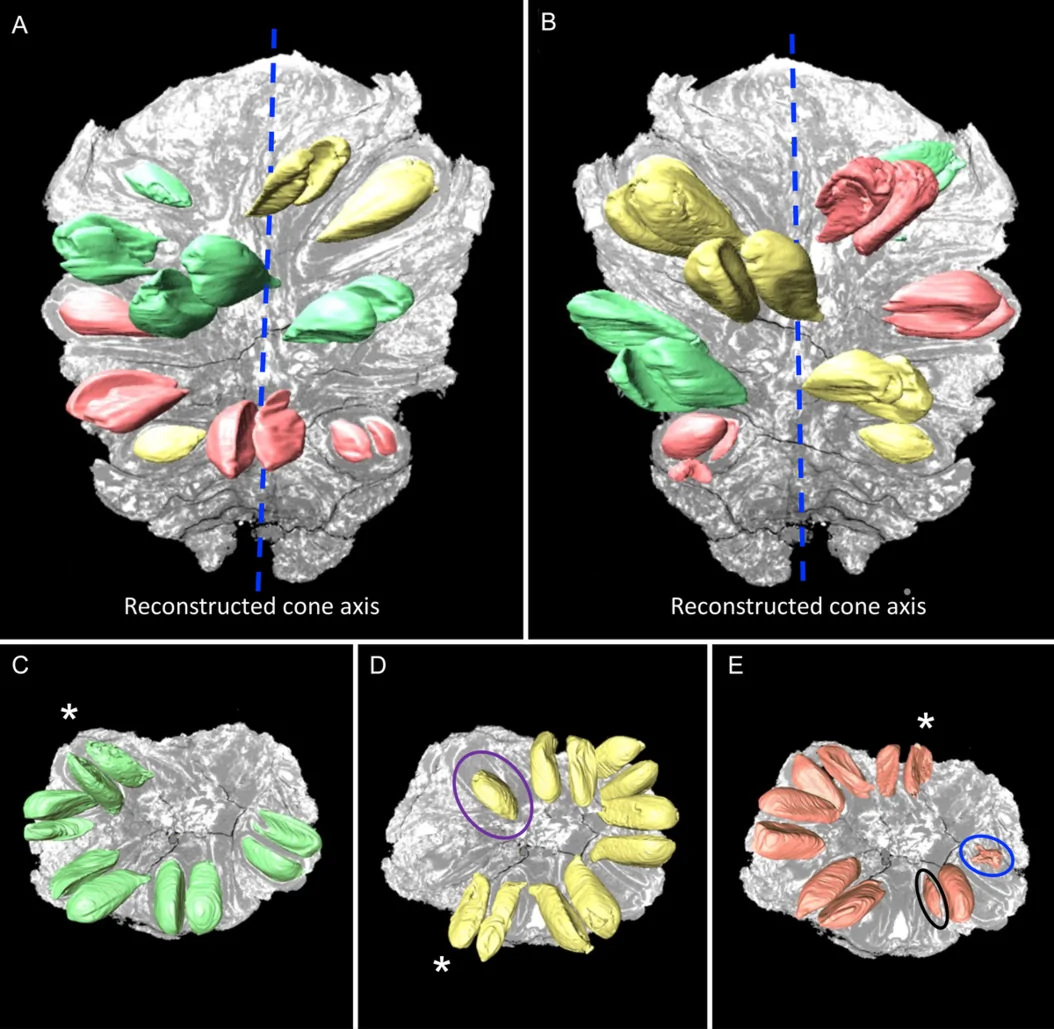
Orthoslices in gray shades imaged with microCT and 3D reconstruction of seed spirals segmented with Avizo, showing the phyllotactic organization of the seeds, and thus of the bract/scale complexes in cone CG‐029 shown in Figure 2A. The long diameter is ca. 17 mm in all cases. (A, B) Three spirals of seeds segmented in different colors within the same cone in two lateral views. The dashed blue line marks the approximate position of the reconstructed polar axis. In this cone, the ontogenetic development of seeds is counterclockwise, from the cone apex to base (see also Appendix S1 for animated version). (C–E) Basal views of the same cone, each highlighting one of the three phyllotactic seed spirals. An asterisk (*) marks the highest seed of each seed spiral. Note that each bract/scale complex bears two seeds, with the exception of the lowermost bract/scale complexes in the yellow and red seed spirals. Circled in purple is a single seed (one seed of the pair did not develop), in black is a seed that is significantly smaller than its partner, and in blue a pair of malformed seeds, the first to form in this seed spiral and in the entire cone.

The seeds are elliptical to broadly obovate in outline (Figures 2A, B, 4A, 5A–E); an average‐sized seed cut along its longitudinal axis in the median longitudinal plane of the cone roughly measures 5 × 2.5 mm. Seeds are positioned at the base of the ovuliferous scale. Two seeds per bract/scale complex can be observed in surface‐abraded cones; both seeds are enclosed together in a pocket‐forming tissue (e.g., CG‐082, Figure 4B).

In orthoslices of cone CG‐029 produced by microCT, each bract/scale complex bears two seeds, both enclosed in a shared pocket‐forming tissue (Figure 4A, arrow). Up to five pairs of seeds can be observed in this transverse section (Figure 4A, asterisks).

In the segmented reconstructions of the orthoslices of cone CG‐029, the lateral view shows two seeds per bract/scale complex and three spirals with seeds (Figure 5A, B). In basal (proximal) view, it becomes evident that two of the seed spirals are incomplete. While the highest seed spiral, the green spiral, has a complete set of five pairs of seeds (Figure 5C), the basalmost bract/scale complex of the yellow spiral bears only one seed, instead of two (Figure 5D, purple circle). Moreover, the basalmost seeds in the red spiral are reduced to a small, fused set of malformed structures (Figure 5E, blue circle), while the second pair of seeds in this spiral has one seed that is much smaller than the other one on the bract/scale complex (Figure 5E, black circle).

#### Phyllotaxis of seeds and cone scales

As is clear in the spiral patterns of the bract/scale complexes on the surface of the cones and through the segmentation of the serial sections produced by microCT, the bract/scale complexes are helically arranged. Contact parastichies that can be observed on the cone surface are 2,3, which is reflected in the number of seed spirals in the cone (Table 2). The degree of rotation of each seed spiral around the cone CG‐029 was at least 305°, which may have been 360° in fully developed cones.

In addition to showing the number of seeds borne by each bract/scale complex as discussed above, the segmented reconstructions of the orthoslices of cone CG‐029 can also be used to trace the ontogenetic spiral of seeds and, hence, bract/scale complexes. This ontogenetic spiral is not one of the two or three phyllotactic spirals; instead, it contains all seeds in the cone. Starting from the uppermost seed pair (here, the most apical pair in the green spiral) and proceeding downward to the next highest pair (the most apical yellow pair) and so on toward the cone base illustrates the order in which they were produced by the apical meristem of the cone (Figure 5A, B). The ontogenetic spiral in cone CG‐029 shows that seed development proceeded counterclockwise, from the cone apex to base.

## DISCUSSION

The seed cones from the Upper Jurassic Morrison Formation of central Utah are assigned here to the genus *Pararaucaria* of the extinct conifer family Cheirolepidiaceae based on several diagnostic features (see also Gee et al., 2014). These include (1) the small, spheroidal to cylindrical shape of the cones with helically arranged bract/scale complexes, (2) a slender cone axis and small‐diameter pith, (3) paired bract and ovuliferous cone scales that are free from one another for the entirety of their lengths, (4) vascular traces of the bract and ovuliferous scale that are also free for nearly the entirety of their respective lengths, (5) the presence of normally two seeds, rarely one, per ovuliferous cone scale, (6) the absence of a seed wing, and (7) the enclosure of each pair of seeds in a shared pocket‐forming tissue. This last character, the occurrence of a pocket‐forming tissue that surrounds the seeds on the ovuliferous cone scale, is considered the most definitive structure characterizing ovulate cones in *Pararaucaria* (Escapa et al., 2012, 2013). As documented in three other species of *Pararaucaria* known from elsewhere in the world, the slender cone peduncle bears short, triangular, helically arranged *Brachyphyllum*‐type scale leaves.

In all specimens of the new species of *Pararaucaria utahensis* established here, the outer surface of the cone is heavily abraded, and the three lobes of the ovuliferous scale sometimes observed in other species is not evident. However, the heavy abrasion to the outside of the *P. utahensis* cones makes it possible to see in some cases the genus‐diagnostic pocket‐forming tissue that covers the pair of seeds on a bract/scale complex.

### Cone phyllotaxis of *Pararaucaria utahensis*

While all *Pararaucaria* species clearly show a helical arrangement of the bract/scale complexes, the phyllotaxis of the bract/scales in *P. utahensis* cones was determined in our study as 2,3. In a concurrent study of *P. patagonica*, it is also 2,3 (M. M. Howell, unpublished data), which seems to be characteristic for the genus. As mentioned earlier, the parastichies in *P. utahensis* are confirmed by the color segmentation of the seed spirals based on microCT orthoslices, which show either three nearly 360° spirals of seeds that wrap around the cone counterclockwise from the cone apex to base (CG‐029, Figure 5A–E), or two spirals of seeds that wrap around the cone clockwise from the cone apex to base.

While the parastichy numbers describe the physical, close packing of the bract/scale complexes in *Pararaucaria utahensis* cones, of greater biological interest is the ontogenetic development of the seeds and bract/scale complexes. In the smallest cone of *Pararaucaria utahensis* (CG‐029), the ontogenetic spiral of seeds (cf. Howell et al., 2026), sometimes called the “genetic spiral” or “generative spiral” (e.g., Mitchinson, 1977; Korn, 2008), shows the directionality of ontogenetic development within this cone. In cone CG‐029 of *P. utahensis*, seed development proceeded counterclockwise, from the cone apex to base, as it does in four more cones of *P. utahensis* (CG‐068, CG‐078, CG‐81, CG‐085; Table 2). Only in one cone (CG‐043) does the ontogenetic development of the seeds appear to have been clockwise from the cone apex to base.

### Comparison of *Pararaucaria* spp.

A tabular comparison highlights both similarities and differences between the seven species of *Pararaucaria* (Table 3). The most evident difference is that each of the seven species has a different geographic and geologic provenance. The three species from Argentina, for example, were described from different rock formations and chronostratigraphic series of the Jurassic in Patagonia: *P. taquetrensis* (Lower Jurassic), *P. patagonica* (Middle Jurassic), and *P. delfueyoi* (Upper Jurassic). Similarly, the two species in the western United States occur in different formations and chronostratigraphic series: *P. carrii* (Middle Jurassic) and *P. utahensis* (Upper Jurassic). The last two species were discovered on continents distant from the others: *P. laiyangensis* (Asia) and *P. collinsonae* (Europe).

**Table 3:** Morphological differences among the seven global species of *Pararaucaria*, organized from geologically oldest to youngest.

Morphological differences	*P. taquetrensis* (4 cones)	*P. patagonica* (100 s of cones)	*P. carrii* (1 cone)	*P. delfueyoi* (16 cones)	*P. utahensis* (12 cones)	*P. collinsonae* (2 cones)	*P. laiyangensis* (6 cones)
Geological series	Lower Jurassic (Pleinsbachian– Toarcian)	Middle Jurassic (Bajocian–Callovian)	Middle Jurassic (Callovian)	Upper Jurassic (Oxfordian/Kimmeridgian boundary)	Upper Jurassic (Kimmeridgian)	Upper Jurassic (Tithonian)	Lower Cretaceous (Hauterivian– Barremian)
Locality	Lonco Trapial Group, Chubut province, Argentina	La Matilde Formation, Santa Cruz province, Argentina	Trowbridge Formation, Oregon, USA	Cañadón Calcáreo Formation, Chubut province, Argentina	Morrison Formation, Utah, USA	Purbeck Limestone Group, England, UK	Laiyang Formation in Shandong, China
Size of cone (cm)	2.3 × 1.2	2.3–5.1 × 1.3–3.0	2.6 × 1.3	Up to 8 × 3–4	1.8–3.0 × 1.7–2.0	1.3 × 1.3	Up to 2.2 × 2.2
Diameter of woody cone axis (mm)	1.5	Slender	ca. 3.75	3.5	4.1	1.0–1.4	2.6
Diameter of pith (mm)	Not given	0.5–0.8	0.75	1.2	2.8	0.3–0.6	Not given
Outline of scale trace after divergence in transverse view	Not given	Inverted U‐shape	Band‐shaped	Hemispherical with a convex adaxial side	Flattened or band‐shaped, very slightly concave down	Inverted U‐shape?	Indet.
Outline of bract trace after divergence in transverse view	Not given	Round	Crescent‐shaped	Circular to subcircular	Circular	Ovoid	Indet.
Distal branching of bract trace	Indet.	Not given, presumably unbranched	Repeatedly	Not given	Largely unbranched, but can abruptly change course	Repeatedly	Indet.
Number of seeds per ovuliferous scale	2	1 (rarely 2)	1	2	2 (rarely 1)	1	1
Seed size (mm)	3.9 × 2.2	6 × 3	5.3 × 6.5	11 × 5	3–4 × 3–4	3–4 × 3–4	3.4 × 1.7
Seed shape	Ellipsoidal	Heart‐shape; widely obovoid	Widest in midregion	Obovoid	Elongate obovoid	Rounded (not cordate)	Oval
Phyllotaxis (clockwise, counterclockwise)	Spiral	Spiral, 2,3	Spiral	Spiral, 2,3^a^	Spiral, 2,3	Spiral, 2,3^a^	Spiral
Organically attached foliage	*Brachyphyllum*	None	None	None	*Brachyphyllum*	None	*Brachyphyllum*
References	Escapa and Leslie (2017)	Wieland (1935); Calder (1953); Stockey (1977); Escapa et al. (2012); M. M. Howell (unpublished data)	Stockey and Rothwell (2013)	Escapa et al. (2013)	Present study	Steart et al. (2014)	Jin et al. (2023)

^a^ New assessment of parastichies based on published photos in original publications by M.M.H.

The stratigraphic occurrences of all species known so far are restricted to the geological time interval between the Lower Jurassic to the Lower Cretaceous, with most species appearing in the Middle to Upper Jurassic. The number of cones found of each species varies from one specimen of *P. carrii* to hundreds of *P. patagonica* cones. The huge abundance of *P. patagonica* makes it possible to carry out statistical work on this species, which may give greater insight into the genus as a whole (e.g., M. M. Howell, unpublished data).

Morphologically, the most striking difference between the *Pararaucaria* cones is the much larger size of *P. delfueyoi*, which is 1.5 to 6 times longer than the others. This larger size is also reflected in the longer size of the seeds of *P. delfueyoi*, which are 1.8 to 3.5 times larger. There is some variation in seed shape in all species, from generally ellipsoidal to cordate, but in our opinion, original seed shape may have been greatly altered by preservation in some species.

One difference that divides *Pararaucaria* species into two groups is the occurrence of one or two seeds on each bract/scale. Four species—*P. patagonica*, *P. carrii*, *P. collinsonae*, and *P. laiyangensis—*have only one seed on each bract/scale, while three species—*P. taquetrensis*, *P. delfueyoi*, and *P. utahensis*—bear two seeds per bract/scale. This character does not seem to be correlated with geological age and thus evolutionary time.

Common to all species is the general outline in cross section of the scale trace after divergence from the cone axis, which varies slightly from band‐shaped to slightly concave‐down to an inverted U. Similarly, the outline of the bract trace after divergence varies slightly, but is, generally speaking, circular in cross section. One puzzling characteristic among three species of *Pararaucaria* is the course of the bract trace after its divergence from the scale trace at the cone axis. In *P. carrii* and *P. collinsonae*, the bract trace is reported as repeatedly branching. In our study of *P. utahensis*, the bract trace appears unbranched, but may abruptly change course soon after divergence, whereas the course of the scale trace is usually smoother and less abrupt.

It should also be noted that the same sort of small, scale leaves of *Brachyphyllum* have been found organically attached to the peduncles of three species (*P. taquetrensis*, *P. utahensis*, and *P. laiyangensis*).

In summary, beyond geographic and geologic origin, *Pararaucaria utahensis* differs from the other species of *Pararaucaria* in morphological details. *Pararaucaria utahensis* differs from *P. delfueyoi* in its much smaller cone and seed size. It differs from *P. patagonica*, *P. carrii*, *P. collinsonae*, and *P. laiyangensis* in bearing two seeds on each bract/scale. Another feature that separates *P. utahensis* from *P. patagonica* is that *P. utahensis* cones have a more spheroid shape, while those of *P. patagonica* are generally more elongated (cf. Stockey, 1977; Escapa et al., 2012; M. M. Howell, unpublished data). *Pararaucaria utahensis* can be differentiated from *P. carrii* and *P. collinsonae* in that its distal bract trace is largely unbranched, while the other two species have a distal bract trace that has been reported as repeatedly branched. The last species‐to‐species comparison, namely between *P. utahensis* and *P. taquetrensis*, shows that the diameter of the woody axis *P. utahensis* (4.1 mm) is significantly larger than that of *P. taquetrensis* (1.5 mm), that is, 2.6 mm wider and thus 11.4 mm^2^ larger in area (86.4%). For these reasons, *P. utahensis* stands on its own as a separate species.

### Paleobiogeography of *Pararaucaria* spp. in the Mesozoic

**Figure 6:**
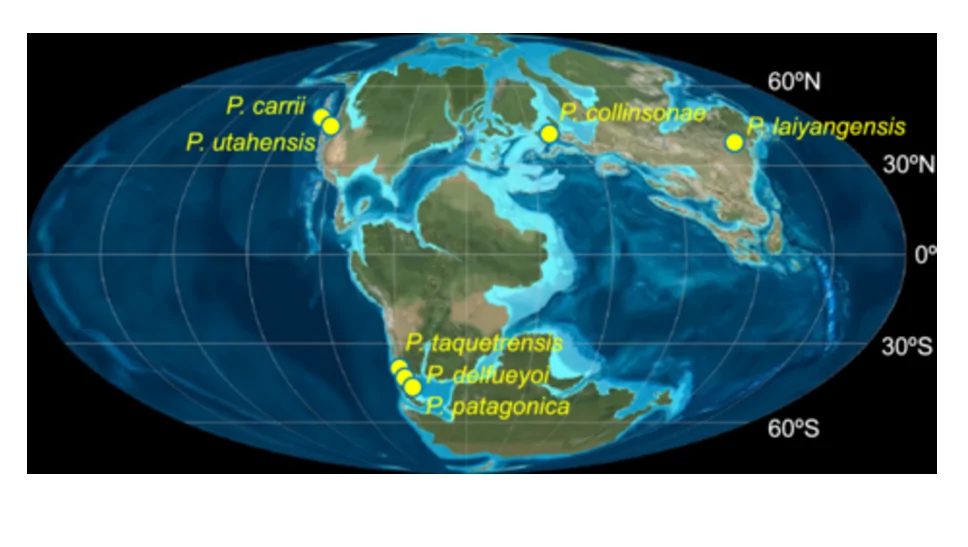
Global paleogeographic map of the Upper Jurassic, marked with the localities of the seven known species of *Pararaucaria*, which occur stratigraphically from the Lower Jurassic to Upper Cretaceous. (Modified from base map by Ron Blakey, Deep Time Maps, https://deeptimemaps.com/map-lists-thumbnails/global-series/).

As previously mentioned, *Pararaucaria* cones have been described from both the northern and southern hemispheres. It is notable that the seven species of *Pararaucaria* have only been reported from the mid‐latitudes of the Mesozoic world, between the paleolatitudes of 30° to 60°N and between 30° to 60°S (Figure 6). They did not occur in the polar regions, nor in the equatorial zone during the Mesozoic. Two species of *Pararaucaria* occur in the western United States (*P. carrii*, *P. utahensis*), one species in England (*P. collinsonae*), one species in northeastern China (*P. laiyangensis*), and three species in what is today Patagonia, Argentina (*P. taquetrensis*, *P. delfueyoi*, and *P. patagonica*).

### Occurrence of Cheirolepidiaceae in the Morrison flora: an enigma

As shown in Figure 1, the occurrence of *Pararaucaria utahensis* cones in the Upper Jurassic Morrison Formation is restricted to one state in the Western Interior of North America. Although cones have been found at four different localities, the area encompassing these sites occupies only a relatively small area in central Utah. In comparison, the pollen of *Classopollis* (syn. *Corollina*), an extremely distinctive sporomorph type associated with the family Cheirolepidiaceae, is found throughout the Morrison Formation in the United States, from Montana in the north, to Arizona and New Mexico in the south (Litwin et al., 1998; Hotton and Baghai‐Riding, 2010). *Classopollis* was found in nearly every sample from the Morrison Formation studied by Hotton and Baghai‐Riding (Carol Hotton, Smithsonian National Museum of Natural History, personal communication), although it is less common in the north and more abundant in the south (Hotton and Baghai‐Riding, 2010). *Classopollis* pollen tends to dominate in the Salt Wash Member (Litwin et al., 1998), particularly in the lower part of the member (Carol Hotton, personal communication).

The widespread and common occurrence of *Classopollis* pollen compared to the extremely limited megafossil evidence of Cheirolepidiaceae in the Morrison Formation is enigmatic. Where and what are the other cheirolepidiaceous megafossils in this geological formation? Up to now, plant megafossils of the family are only represented by the *Pararaucaria* seed cones described here.

It is conceivable that cheirolepidiaceous megafossils have already been described from the Morrison Formation, but not yet formally recognized as pertaining to this family. One possible candidate is *Brachyphyllum rechteniorum*,^1^ a branch bearing several rhomboidally shaped, persistent, appressed leaves with cuticle (Tidwell et al., 1998). The cuticle possesses sunken pits around the stomata formed by a ring of commonly five subsidiary cells each bearing papillae that are either upright or overarching the stomatal chamber. Such a stomatal pit surrounded by subsidiary cells with papillae is characteristic of cheirolepidiaceous cuticle (e.g., Alvin, 1982; Watson, 1988).

The characters that prevented Tidwell et al. (1998) from attributing *Brachyphyllum rechteniorum* to the Cheirolepidiaceae, such as a relatively thinner cuticle and the arrangement of stomata in regular longitudinal rows instead of being scattered, may turn out not to be such important features for exclusion; for example, other cheirolepidiaceous leaves are now known to have stomata arranged in longitudinal bands (e.g., Tosolini et al., 2015). Note, that while ginkgophyte leaves with cuticle occurring in the Morrison Formation possess papillae (C. T. Gee and J. Foster, Utah Field House of Natural History State Park Museum, unpublished data), their leaf form is extremely different from *Brachyphyllum*, a leaf type associated with conifers such as Araucariaceae, Cheirolepidiaceae, Cupressaceae, and Podocarpaceae (Barnard and Miller, 1976). The cuticle of living species in Araucariaceae (Stockey and Ko, 1986; Stockey and Atkinson, 1993; Chambers et al., 1998) and Podocarpaceae (Stockey and Ko, 1988, 1990; Stockey and Frevel, 1997; Stockey et al., 1992, 1995, 1998; Mill and Schilling, 2009) does not have papillae.

Another possible plant megafossil pertaining to the Cheirolepidiaceae is a new, still undescribed leafy conifer twig from the Jurassic Salad Bar flora in the Upper Jurassic Morrison Formation near Blanding, Utah. Designated as “Morphotype 1,” this twig bears distinctive leaves with a clasping base and a lamina that splits into narrow, longitudinally aligned segments (Foster et al., 2023, figure 1l); when detached from the wood axis, the individual leaves are spade‐shaped with a curved base where it clasps around the twig (C. T. Gee, unpublished data). Similar twigs with scale‐like leaves with series of longitudinal ridges and grooves and a clasping base, which were called *Araucarities sanctaecrucis*, have been found attached to the peduncle of *Pararaucaria patagonica* seed cones in the Middle Jurassic La Matilde Formation in Patagonia, Argentina (Falaschi et al., 2011, figure 12).

A number of other types of clearly coniferous foliage found in the Upper Jurassic Morrison Formation could also pertain to the Cheirolepidiaceae, such as *Pagiophyllum*, *Cupressinocladus*, *Podozamites*, *Pityophyllum*, *Pityospermum*, and *Elatides* (Ash and Tidwell, 1998). It is certain that further paleobotanical work in the Morrison Formation with an eye open for members of the Cheirolepidiaceae will help to resolve this puzzle.

## CONCLUSIONS

A new species of *Pararaucaria* seed cone, *P. utahensis* Gee, Howell et Dayvault sp. nov., is described here from the Upper Jurassic of the Morrison Formation of Utah, United States, based on the remains of 12 specimens. In addition to the gross morphology of the cones, their internal construction was studied nondestructively using microCT and integrated 3D visualization. In nearly all cases, each bract/scale complex bears two seeds enclosed in the pocket‐forming tissue that is diagnostic for the genus. Another key characteristic is separation of the bract and scale and of the bract and scale traces at their divergence from the cone axis. The cone axis is slender and contains a narrow‐diameter pith. Phyllotaxis of the bract/scale complexes and of the seeds is 2,3, while the ontogenetic development of the bract/scale complexes and seeds is generally counterclockwise. Small, imbricate, *Brachyphyllum*‐type scale leaves are borne on the cone peduncle. *Pararaucaria utahensis* is the seventh global species of *Pararaucaria* and the second species in North America. Until now, it is the only unequivocal megafossil evidence for the conifer family Cheirolepidiaceae in the Upper Jurassic Morrison Formation of western North America. Because of the widespread occurrence of cheirolepidiaceous pollen throughout this formation, the absence of other plant megafossils clearly pertaining to this family remains an enigma.

## AUTHOR CONTRIBUTIONS

C.T.G. and R.D.D. designed the study and collected the fossil material from the field. C.T.G. gathered the cone data in the lab. C.T.G. and M.M.H. carried out the microCT scans; M.M.H. did the segmentation. C.T.G. described the cones, interpreted the data with support from M.M.H., produced the figures, and wrote the manuscript. C.T.G., M.M.H., and Jalena Dayvault on behalf of R.D.D. approved the final version of the paper.

## CONFLICT OF INTEREST STATEMENT

Carole T. Gee is an Associate Editor of the *American Journal of Botany*, but took no part in the peer‐review and decision‐making processes for this paper.

## Supporting information


**Appendix S1:** Video of 3D reconstruction of the three seed spirals segmented in different colors to show the phyllotactic organization of the seeds in lateral view in cone CG‐029. The ontogenetic development of each spiral of seeds starts at the apical meristem and proceeds counterclockwise down the cone.

**Appendix S2:** Video of 3D segmented cone axis and vascular traces in cone CG‐068, showing the slender central cone axis and the two separate traces originating from the axis and extending into each bract/scale complex. The ontogenetic development of bract/scale traces starts at the apical meristem and proceeds counterclockwise down the cone.

**Appendix S3:** Video of 3D segmented cone axis and vascular traces in cone CG‐078, showing the slender central cone axis and the two separate traces originating from the axis and extending into each bract/scale complex. The ontogenetic development of bract/scale traces starts at the apical meristem and proceeds counterclockwise down the cone.

## Data Availability

The microCT *x*‐*y* and *x*‐*z* image stacks of cone CG‐029 used in this study are deposited in Figshare: https://doi.org/10.6084/m9.figshare.33023477.

## Footnotes

1 In accordance with Article 60.8 of the *International Nomenclature for fungi, algae, and plants* (Turland et al., 2018), the original species epithet *B. rechtenii* is altered here to *B. rechteniorum*, which would then have the correct orthographical ending meant for Mr. and Mrs. Paul Rechten of Harvard, Illinois (Tidwell et al., 1998).
